# Identification of Proteomic Signatures in Chronic Obstructive Pulmonary Disease Emphysematous Phenotype

**DOI:** 10.3389/fmolb.2021.650604

**Published:** 2021-07-01

**Authors:** Shuang Bai, Rui Ye, Cuihong Wang, Pengbo Sun, Di Wang, Yong Yue, Huiying Wang, Si Wu, Miao Yu, Shuhua Xi, Li Zhao

**Affiliations:** ^1^ Department of Pulmonary and Critical Care Medicine, Shengjing Hospital of China Medical University, Shenyang, China; ^2^ Department of Radiology, Shengjing Hospital of China Medical University, Shenyang, China; ^3^ Department of Biobank, Shengjing Hospital of China Medical University, Shenyang, China; ^4^ Department of Environmental and Occupational Health, School of Public Health, China Medical University, Shenyang, China

**Keywords:** chronic obstructive pulmonary disease, emphysematous phenotype, proteomics, KRT17, DHRS9

## Abstract

Chronic obstructive pulmonary disease (COPD) is a highly heterogeneous disease. Emphysematous phenotype is the most common and critical phenotype, which is characterized by progressive lung destruction and poor prognosis. However, the underlying mechanism of this structural damage has not been completely elucidated. A total of 12 patients with COPD emphysematous phenotype (COPD-E) and nine patients with COPD non-emphysematous phenotype (COPD-NE) were enrolled to determine differences in differential abundant protein (DAP) expression between both groups. Quantitative tandem mass tag–based proteomics was performed on lung tissue samples of all patients. A total of 29 and 15 lung tissue samples from patients in COPD-E and COPD-NE groups, respectively, were used as the validation cohort to verify the proteomic analysis results using western blotting. Gene ontology (GO) and Kyoto Encyclopedia of Genes and Genomes (KEGG) enrichment analyses were conducted for DAPs. A total of 4,343 proteins were identified, of which 25 were upregulated and 11 were downregulated in the COPD-E group. GO and KEGG analyses showed that wound repair and retinol metabolism–related pathways play an essential role in the molecular mechanism of COPD emphysematous phenotype. Three proteins, namely, KRT17, DHRS9, and FMO3, were selected for validation. While KRT17 and DHRS9 were highly expressed in the lung tissue samples of the COPD-E group, FMO3 expression was not significantly different between both groups. In conclusion, KRT17 and DHRS9 are highly expressed in the lung tissue of patients with COPD emphysematous phenotype. Therefore, these proteins might involve in wound healing and retinol metabolism in patients with emphysematous phenotype and can be used as phenotype-specific markers.

## Introduction

Chronic obstructive pulmonary disease (COPD) emphysematous phenotype is characterized by lung tissue destruction and distal airspace over inflation (Alford et al., 2010; Camp et al., 2014; Wang et al., 2018). In contrast to patients with other COPD phenotypes, those with emphysematous phenotype typically manifest symptoms such as severe dyspnea, decreased exercise tolerance, and physiological complications. Moreover, patients with emphysematous phenotype have a higher mortality than those with non-emphysematous phenotype (Fan et al., 2014; Criner et al., 2018; Bai et al., 2020). Some emphysematous smokers do not exhibit airflow limitation (Oelsner et al., 2014), despite showing similar pathophysiological features to those with COPD (Bai et al., 2020). Unfortunately, there are few biomarkers that could be useful to predict the emphysema phenotype in COPD patients. And pharmaceutical intervention to reverse lung destruction or inhibit pulmonary damage is not available for these patients (Vogelmeier et al., 2017; Janssen et al., 2019).

Low blood eosinophils, elevated plasmin–mediated degradation of cross-linked fibrin, and hypermethylation of microRNA-7 are regarded as the features of emphysematous phenotype (Papaioannou et al., 2017; Manon-Jensen et al., 2019; Rosas-Alonso et al., 2020), but their potential to serve as prognostic biomarkers needs further validation. Moreover, the underlying molecular mechanism of COPD emphysematous phenotype remains unclear. Besides, imbalances between proteases/antiproteases and oxidants/antioxidants, enhanced inflammatory response (Lan et al., 2019; Zeng et al., 2020), and dysfunctional tissue repair in the lung parenchyma and interstitium play an important role in the pathogenetic mechanisms of emphysema (Jiang et al., 2017; Belgacemi et al., 2020; Hu et al., 2020). However, the underlying mechanisms remain poorly understood.

Proteomics reflects proteins expression changes, which are occurring in organisms. It is an important tool to understand pathogenesis and identify disease biomarkers. Tandem mass tag (TMT) quantitative proteomics is a reliable approach to measure protein content, and it has been increasingly used to identify pathogenetic mechanisms and human disease biomarkers (Mertins et al., 2018; Dziekan et al., 2020; Wang et al., 2020). While most proteomic studies in patients with COPD have used bronchoalveolar lavage fluid (Tu et al., 2014), sputum (Titz et al., 2015), and blood (Sridhar et al., 2019), only a few have used lung tissue samples. Furthermore, as per our understanding, the studies that specifically addressed COPD emphysematous phenotype are not available. Since COPD is a highly heterogeneous disease, the proteomic analysis focused on its emphysematous phenotype has remarkable significance.

In this study, we conducted proteomic analysis of lung tissue samples using TMT quantitative proteomic techniques to identify key molecules and their underlying mechanisms in patients with COPD emphysematous phenotype.

## Materials and Methods

### Patients

Patients with COPD were recruited based on the criteria previously described (Bai et al., 2020). In brief, the inclusion criteria were as follows: 40- to 80-year-old patients diagnosed with COPD, more than 10 pack-years of smoking history, preparing for chest surgery, and read and signed informed consent. Patients with other respiratory diseases, autoimmune diseases, or infections that occurred within 4 weeks before surgery were excluded. All enrolled patients were assigned to two groups: the COPD emphysematous phenotype (COPD-E) group and the COPD non-emphysematous phenotype (COPD-NE) group, according to the diagnosis of investigators and results of image analysis. Chest computed tomography (CT) scans revealed obvious emphysema in COPD-E patients and almost no emphysema in COPD-NE patients. The patients in both groups were matched for sex, age, and smoking history. The study protocol was approved by the Ethics Committee of Shengjing Hospital of China Medical University (Shenyang, China; Ethical no. 2016PS342K). For each patient included, written informed consent was obtained.

### CT Scanning

Chest CT scans were obtained for all patients using Philips Brilliance iCT 256 (Philips, Surrey, United Kingdom), TOSHIBA Aquilion ONE (Toshiba, Tokyo, Japan), Siemens SOMATOM Definition Flash (Siemens, Forchheim, Germany), or GE Optima CT660 (GE, Milwaukee, WI, United States). All scans were obtained at a deep inspiratory breath hold. The following parameters were applied in the examination: tube voltage, 120 kV; tube current, 180 mA; and reconstruction matrix, 512 × 512. Two radiologists performed radiological measurements. The detailed CT acquisition parameters are shown inSupplementary Table S1.

### Emphysema Screening

Chest CT scans of the patients were acquired from the CT workstation (Neusoft, Shenyang, China), and the image analyses were performed using Pulmonary Toolkit in Matlab (R2016a) (The MathWorks, Inc., Natick, MA, United States). The degree of emphysema was determined quantitatively according to our previous study (Bai et al., 2020). In brief, voxels with a CT value less than −950 HU were identified as the emphysematous area. The emphysema index was defined as the lung volume fraction of voxels with a CT value below −950 HU at a full inspiration (Camp et al., 2009; Coxson et al., 2014). The emphysema percentile density was defined as the 15th percentile lung density derived from the distribution histogram of whole lung CT voxel (Dirksen et al., 2009). The raw data of all CT images were entered into the analysis software, following which the results were obtained.

### Sample Preparation

Samples were prepared as described previously (Sun et al., 2019; Bai et al., 2020). In brief, lung tissue samples were obtained as far as possible from the tumor (at least 5 cm from the boundary). Lung parenchyma, pulmonary vessels, and small airways were equal approximately in each sample. Each specimen was cleaned with normal saline and dried. The collected lung tissue samples were frozen with liquid nitrogen immediately and stored at −80°C until use.

The lung tissue samples were lysed using the SDT (4% SDS (w/v); 0.1 M DTT; 100 mM Tris-HCl, pH 7.6) lysis method. The BCA method was used to determine protein concentration. Every three tissue samples of COPD-NE patients were pooled together to form the three biological replicates in the COPD-NE group, and every four tissue samples of COPD-E patients were pooled together to form the three biological replicates in the COPD-E group. The filter-aided proteome preparation method was used to perform protein collection (Wiśniewski et al., 2009). In brief, lysates were mixed with 8 M urea in the filter unit of Microcon devices (Millipore, Bedford, MA, United States) and centrifuged to remove the low-molecular-weight material. Subsequently, 0.05 M iodoacetamide in 8 M urea was added to the concentrate followed by centrifugation. The resulting concentrate was diluted with 8 M urea and concentrated again. Finally, endoproteinase was added to digest proteins, and the digests were centrifuged to collect peptides. The concentration of peptide was measured at OD280. An equal amount of peptide was used from each sample, and all peptides were labeled using the TMT10 labeling kit (Thermo Fisher Scientific, Waltham, MA, United States) as described in the manufacturer’s instructions. Then, the labeled peptides were mixed, and the high-pH reversed-phase peptide fractionation kit was used to perform grading subsequently. Briefly, the fractionation column was equilibrated with acetonitrile and 0.1% trifluoroacetic acid, and the labeled peptides were loaded into the column. After adding pure water, the samples were centrifuged at a low speed for desalination treatment. Finally, the column-bound peptides were gradient-eluted with increasing concentrations of high-pH acetonitrile solutions. Each eluted peptide sample was dried in vacuum and reconstituted in 0.1% formic acid. The peptide concentration of each sample was measured at OD280.

### Liquid Chromatography-Tandem Mass Spectrometry Analysis

A high-performance liquid chromatography liquid-phase system (Thermo Fisher Scientific, Waltham, MA, United States) was used to separate the graded samples passed consecutively through the loading and analytical columns at 300 nl/min. Subsequently, a Q Exactive mass spectrometer (Thermo Fisher Scientific, Waltham, MA, United States) was used to perform LC-MS/MS analysis. The following parameters were applied for detection: positive ion mode; the precursor ion scanning range was 300–1800 m/z; the first-order mass spectrum resolution was 70,000 at 200 m/z; the automatic gain control target was 1e6; maximum IT at 50 ms; and dynamic exclusion at 60 s. The collection method of peptide fragments’ mass-to-mass ratio was as follows: 20 fragment spectra (MS2 scans) were collected after each full scan; the MS2 activation type was high-energy collision dissociation; and the isolation window was 2 m/z. In addition, the secondary MS resolution was 35,000 at 200 m/z, with a normalized collision energy of 30 eV and 0.1% underfill.

### Protein Identification and Quantification

Proteins were identified in the UniProt database using Mascot 2.2 and Proteome Discoverer 1.4 software. The selection criteria for the trusted peptides were as follows: FDR < 0.01; peptide mass of ±20 ppm; and fragment mass tolerance of 0.1 Da. Protein quantification was performed in accordance with only the unique peptides’ median of the protein. By calculating the relative expression ratio of the target protein to the reference protein for all samples in each group, the protein abundant differences between the two groups were compared. Finally, all peptide ratios were normalized using the median protein ratio.

### Bioinformatics Analysis

**FIGURE 1:**
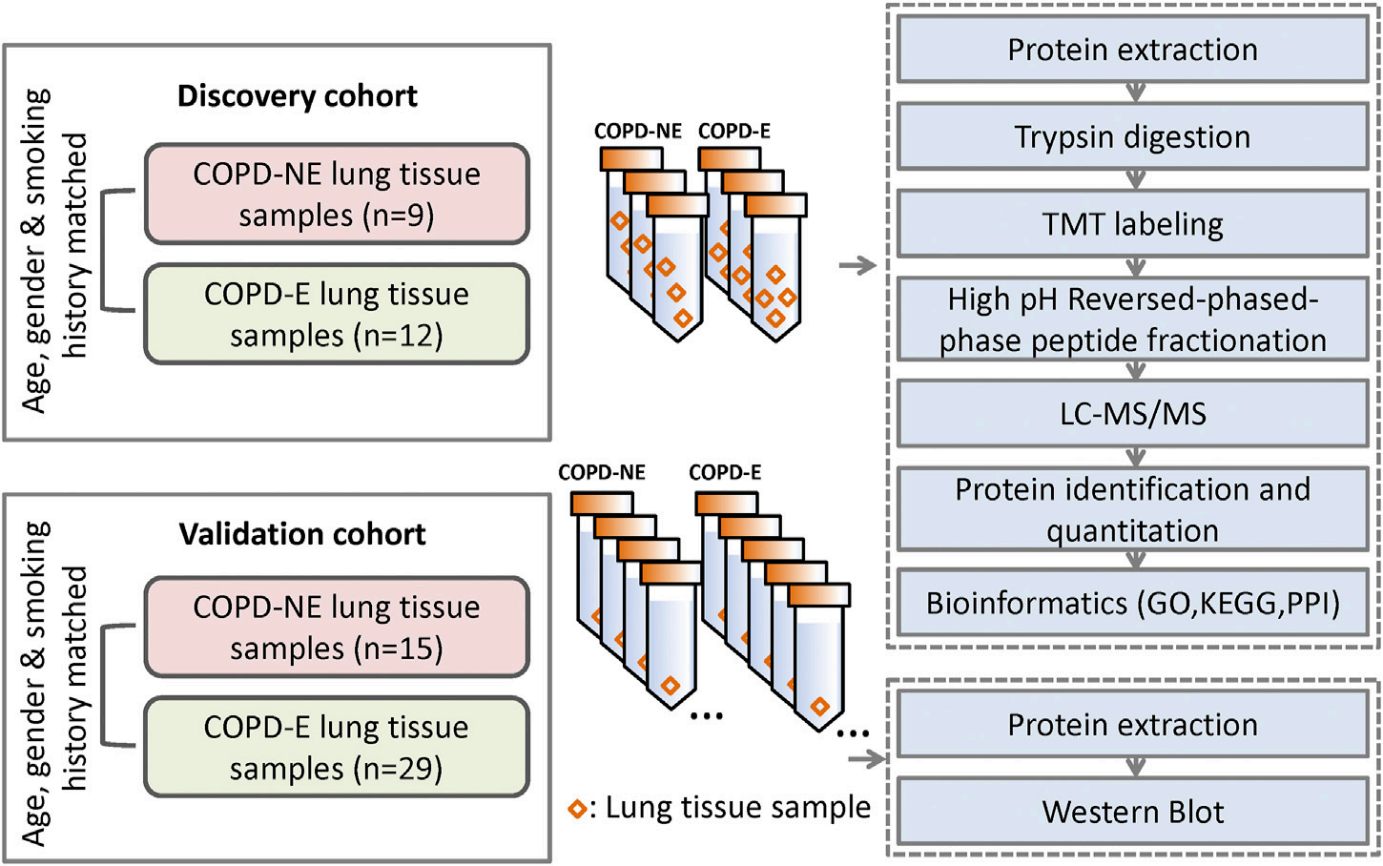
Schematic diagram of tandem mass tag–based quantitative proteomic analysis**.** For the discovery cohort, every three tissue samples of COPD-NE patients were pooled together to form the three biological replicates in the COPD-NE group, and every four tissue samples of COPD-E patients were pooled together to form the three biological replicates in the COPD-E group. Liquid chromatography-tandem mass spectrometry analysis was performed for the discovery cohort. For the validation cohort, every lung tissue sample was used as an independent one. Western blot was performed to validate the results of liquid chromatography-tandem mass spectrometry analysis.

Gene ontology (GO) and Kyoto Encyclopedia of Genes and Genomes (KEGG) pathways were analyzed using Blast2GO and KEGG automatic annotation server software, respectively. Fisher’s exact test was used to compare the distribution of target proteins in the GO and KEGG pathways and perform enrichment analysis. Protein cluster analysis was conducted using the ComplexHeatmap R package (R version 3.4). The expression matrix of target proteins was normalized prior to the generation of the hierarchical cluster heat map. Protein interaction network analysis of target proteins was based on the STRING database and visualized with Cytoscape software (version 3.6.0). The overall scheme of TMT quantitative proteomics is shown in Figure 1.

### Western Blot Analysis

Western blotting was performed according to our previous protocols (Bai et al., 2020). In brief, total protein extracts were prepared using RIPA lysis buffer, and the concentration of protein was estimated using the BCA method. In sodium dodecyl sulfate–polyacrylamide gel electrophoresis, equal amounts of denatured proteins were loaded. Each sample protein was transferred from gel to a polyvinylidene fluoride membrane, blocked with 5% non-fat milk, and incubated overnight with the primary antibody at 4°C. On the following day, secondary antibodies were added to incubate the membrane for 1 h. For chemiluminescence detection, protein bands were revealed using enhanced chemiluminescence reagent. The following primary antibodies were used: anti-KRT17 (ab51056) and anti-FMO3 (ab126711) from Abcam (Cambridge, Cambridgeshire, United Kingdom) and anti-DHRS9 and anti-GAPDH from Proteintech (Chicago, IL, United States).

### Statistical Analysis

All statistical analyses were performed using GraphPad Prism 7 (GraphPad, La Jolla, CA, United States), except for bioinformatics analysis. Bioinformatics-related statistical software and methods are described in *Bioinformatics Analysis*. Student’s *t*-test was used to compare means between two groups of normally distributed data. For data with non-normal distribution, the Mann–Whitney U test was used. The comparison of two groups of categorical variable data was analyzed by Fisher’s exact probability. Continuous variables are expressed as the mean ± standard error of mean. Linear regression analysis was conducted for regression and correlation analyses. *p* < 0.05 was regarded as statistically significant.

## Results

### Demographic and Radiological Features

In total, 16 patients in the COPD-NE group and 29 patients in the COPD-E group were recruited between September 2016 and June 2019. Lung tissue samples of 9 patients in the COPD-NE group and 12 patients in the COPD-E group were used for proteomic analysis. These 21 lung tissues were collected between September 2016 and March 2018 and defined as the discovery cohort. Until June 2019, all samples had been collected and defined as the validation cohort. The validation cohort consisted of 15 COPD-NE lung tissues and 29 COPD-E lung tissues, of which 8 COPD-NE specimens and 12 COPD-E specimens were involved in the discovery cohort. For general features and pulmonary function parameters, there were no significant differences between both groups in the discovery and validation cohorts. However, both cohorts predominantly consisted of male patients, and the discovery cohort consisted of only male patients. In the validation cohort, patients of the COPD-E group had a longer smoking history than those in the COPD-NE group. However, there were no significant differences in pack-years between the two groups. One patient in the discovery cohort and four in the validation cohort did not have pulmonary function test results after bronchodilator administration (Table 1, Table 2).

**TABLE 1:** Demographic and spirometric features of the discovery cohort.

	COPD-NE	COPD-E	*p* value
Patients, *n*	9	12	—
Age, years	62.11 ± 2.80	65 ± 1.94	0.39
Gender	—	—	1.00
Male, *n* (%)	9 (100.0)	12 (100.0)	
Female, *n* (%)	0	0	—
Smokers	—	—	0.12
Current, *n* (%)	5 (55.6)	11 (91.7)	—
Ex-smoker, *n* (%)	4 (44.4)	1 (8.3)	—
Years of smoking	37.78 ± 4.26	42.75 ± 2.45	0.30
Pack-years	36.11 ± 9.64	46.71 ± 5.59	0.33
Post-bronchodilator (COPD-NE, *n* = 8; COPD-E, *n* = 12)			
FEV1 (%)	67.91 ± 7.10	68.29 ± 5.55	0.97
FEV1/FVC	60.89 ± 3.20	57.22 ± 2.58	0.38

Data represent the mean ± SEM of demographic and spirometric features of all patients in each group; COPD, chronic obstructive pulmonary disease; FEV1, forced expiratory volume in 1 s; FVC, forced vital capacity.

**TABLE 2:** Demographic and spirometric features of the validation cohort.

	COPD-NE	COPD-E	*p* value
Patients, *n*	15	29	—
Age, years	62.33 ± 1.90	64.41 ± 1.27	0.36
Gender	—	—	0.60
Male, *n* (%)	13 (86.67)	27 (93.10)	—
Female, *n* (%)	2 (13.33)	2 (6.90)	—
Smokers	—	—	0.41
Current, *n* (%)	11 (73.33)	25 (86.21)	—
Ex-smoker, *n* (%)	4 (26.67)	4 (13.79)	—
Years of smoking	33.67 ± 3.10	40.28 ± 1.76	0.05
Pack-years	41.00 ± 6.51	43.29 ± 3.50	0.74
Post-bronchodilator (COPD-NE, *n* = 13; COPD-E, *n* = 27)			
FEV1 (%)	69.75 ± 4.84	72.25 ± 3.25	0.64
FEV1/FVC	62.17 ± 2.06	60.12 ± 1.57	0.41

Data represent the mean ± SEM of demographic and spirometric features of all patients in each group; COPD, chronic obstructive pulmonary disease; FEV1, forced expiratory volume in 1 s; FVC, forced vital capacity.

In the discovery cohort, patients in the COPD-E group showed a higher emphysema index (2.94 ± 1.12 vs. 11.56 ± 2.78, *p* = 0.02) and lower emphysema percentile density (−911.80 ± 8.30 vs. −944.60 ± 8.40, *p* = 0.01) than those in the COPD-NE group. This result suggested that COPD-E patients had more severe emphysema lesions than COPD-NE patients (Table 3). Other airway-related parameters, including proportion of air and tissue, were not significantly different between both groups. The CT scan results did not show large differences between the validation and discovery cohorts. Raw radiological data were not available for one patient in the validation cohort; this patient was not included in this analysis.

**TABLE 3:** Radiological parameters of the discovery and validation cohorts.

	COPD-NE	COPD-E	*p* value
Discovery cohort			
Patients, *n*	9	12	—
% of air	82.72 ± 1.57	84.46 ± 0.79	0.30
Volume of air (cm^3^)	2,2419 ± 274.50	2,2635 ± 175.30	0.57
% of tissue	17.28 ± 1.57	15.54 ± 0.79	0.30
Volume of tissue (cm^3^)	467.10 ± 27.20	465.50 ± 22.61	0.96
Emphysema index (%)	2.94 ± 1.12	11.56 ± 2.78	0.02^*^
Emphysema percentile density (HU)	−911.80 ± 8.30	−944.60 ± 8.40	0.01^*^
Mean airway lumen radius (mm)	7.02 ± 0.27	6.50 ± 0.28	0.21
Mean airway wall thickness (mm)	3.11 ± 0.21	3.65 ± 0.28	0.16
Ratio of mean airway wall thickness to mean radius	0.44 ± 0.037	0.57 ± 0.06	0.10
Validation cohort			
Patients, *n*	15	28^a^	—
% of air	80.59 ± 1.38	83.00 ± 0.78	0.11
Volume of air (cm^3^)	2090 ± 229.70	2,2372 ± 145.30	0.28
% of tissue	19.41 ± 1.38	11.04 ± 0.78	0.11
Volume of tissue (cm^3^)	448.10 ± 21.68	454.70 ± 17.71	0.82
Emphysema index (%)	4.80 ± 1.31	10.93 ± 1.66	0.02^*^
Emphysema percentile density (HU)	−912.50 ± 7.55	−936.60 ± 5.25	0.01^*^
Mean airway lumen radius (mm)	6.68 ± 0.27	6.55 ± 0.21	0.70
Mean airway wall thickness (mm)	3.47 ± 0.22	3.69 ± 0.22	0.53
Ratio of mean airway wall thickness to mean radius	0.53 ± 0.06	0.56 ± 0.03	0.62

Data represent the mean ± SEM of radiological parameters for all patients in each group.

a Raw radiological data were not available for one patient in the COPD-E group of the validation cohort. COPD, chronic obstructive pulmonary disease.

^*^ *p* < 0.05.

### Protein Identification and Differential Abundant Protein Screening

**FIGURE 2:**
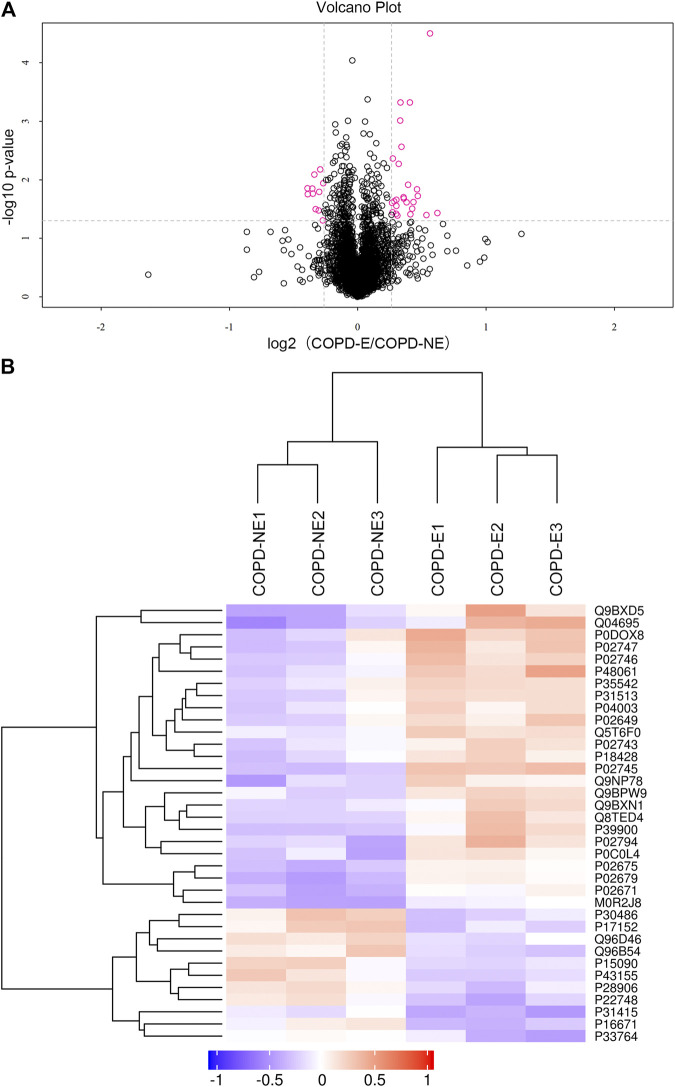
Quantification and hierarchical clustering of differential abundant proteins (DAPs) in COPD emphysmetous phenotype. **(A)** Volcano plot of DAPs. **(B)** Hierarchical clustering heat map of DAPs.

A total of 31,479 peptides, including 27,157 unique peptides, were identified by mass spectrometry, and 4,343 proteins were finally identified. All identified proteins and related information are shown inSupplementary Table S2. The mass spectrometry proteomics data have been deposited to the ProteomeXchange Consortium (http://proteomecentral.proteomexchange.org) *via* the iProX partner repository with the dataset identifier PXD023900 (Ma et al., 2019). DAPs were identified using the following screening criteria: COPD-E/COPD-NE ratio greater than 1.2-fold (upregulation greater than 1.2-fold or downregulation less than 0.83-fold) and *p* < 0.05. A total of 36 DAPs were identified, of which 25 were upregulated and 11 were downregulated. The type I cytoskeletal 17 (KRT17) protein expression in the COPD-E group was 1.54-fold higher than that in the COPD-NE group, as the protein with the most obvious differences between the two groups. All DAPs are listed in Table 4, and a volcano plot showing the distribution of DAPs is illustrated in Figure 2A. Hierarchical cluster analysis showed that the samples were clustered into two categories, which were the same groups as the original groups. The clustering results are shown in Figure 2B.

**TABLE 4:** Differential abundant proteins in the COPD-E and COPE-NE groups.

Accession	Description	Gene name	COPD-E/COPD-NE ratio	*p* value
Q04695	Keratin, type I cytoskeletal 17	KRT17	1.54	0.037^*^
P02745	Complement C1q subcomponent subunit A	C1QA	1.48	<0.001^***^
Q9BXD5	N-acetylneuraminate lyase	NPL	1.45	0.040^*^
P02794	Ferritin heavy chain	FTH1	1.38	0.019^*^
P48061	Stromal cell–derived factor 1	CXCL12	1.38	0.015^*^
P39900	Macrophage metalloelastase	MMP12	1.35	0.024^*^
P02747	Complement C1q subcomponent subunit C	C1QC	1.34	0.031^*^
P0DOX8	Immunoglobulin lambda-1 light chain	—	1.33	0.039^*^
P02679	Fibrinogen gamma chain	FGG	1.32	<0.001^***^
P02746	Complement C1q subcomponent subunit B	C1QB	1.31	0.012^*^
Q9NP78	ATP-binding cassette sub-family B member 9	ABCB9	1.30	0.024^*^
Q8TED4	Glucose-6-phosphate exchanger SLC37A2	SLC37A2	1.28	0.021^*^
P0C0L4	Complement C4-A	C4A	1.28	0.020^*^
P02671	Fibrinogen alpha chain	FGA	1.27	0.003^**^
M0R2J8	Doublecortin domain–containing protein 1	DCDC1	1.26	<0.001^***^
P02675	Fibrinogen beta chain	FGB	1.26	0.001^**^
Q9BPW9	Dehydrogenase/reductase SDR family member 9	DHRS9	1.25	0.005^**^
P02649	Apolipoprotein E	APOE	1.24	0.041^*^
P31513	Dimethylaniline monooxygenase [N-oxide–forming] 3	FMO3	1.23	0.022^*^
P18428	Lipopolysaccharide-binding protein	LBP	1.23	0.028^*^
Q9BXN1	Asporin	ASPN	1.23	0.038^*^
P02743	Serum amyloid P-component	APCS	1.22	0.023^*^
Q5T6F0	DDB1- and CUL4-associated factor 12	DCAF12	1.21	0.004^**^
P04003	C4b-binding protein alpha chain	C4BPA	1.21	0.036^*^
P35542	Serum amyloid A-4 protein	SAA4	1.20	0.025^*^
Q96D46	60S ribosomal export protein NMD3	NMD3	0.83	0.011^*^
P33764	Protein S100-A3	S100A3	0.83	0.049^*^
P31415	Calsequestrin-1	CASQ1	0.82	0.007^**^
P28906	Hematopoietic progenitor cell antigen CD34	CD34	0.81	0.016^*^
P15090	Fatty acid–binding protein, adipocyte	FABP4	0.81	0.033^*^
P43155	Carnitine O-acetyltransferase	CRAT	0.80	0.032^*^
P16671	Platelet glycoprotein 4	CD36	0.79	0.008^**^
Q96B54	Zinc finger protein 428	ZNF428	0.78	0.017^*^
P22748	Carbonic anhydrase 4	CA4	0.78	0.014^*^
P17152	Transmembrane protein 11, mitochondrial	TMEM11	0.76	0.018^*^
P30486	HLA class I histocompatibility antigen, B-48 alpha chain	HLA-B	0.76	0.014^*^

*p < 0.05, **p < 0.01, ***p < 0.001.

### Functional Enrichment and Protein–Protein Interaction Network of DAPs

**FIGURE 3:**
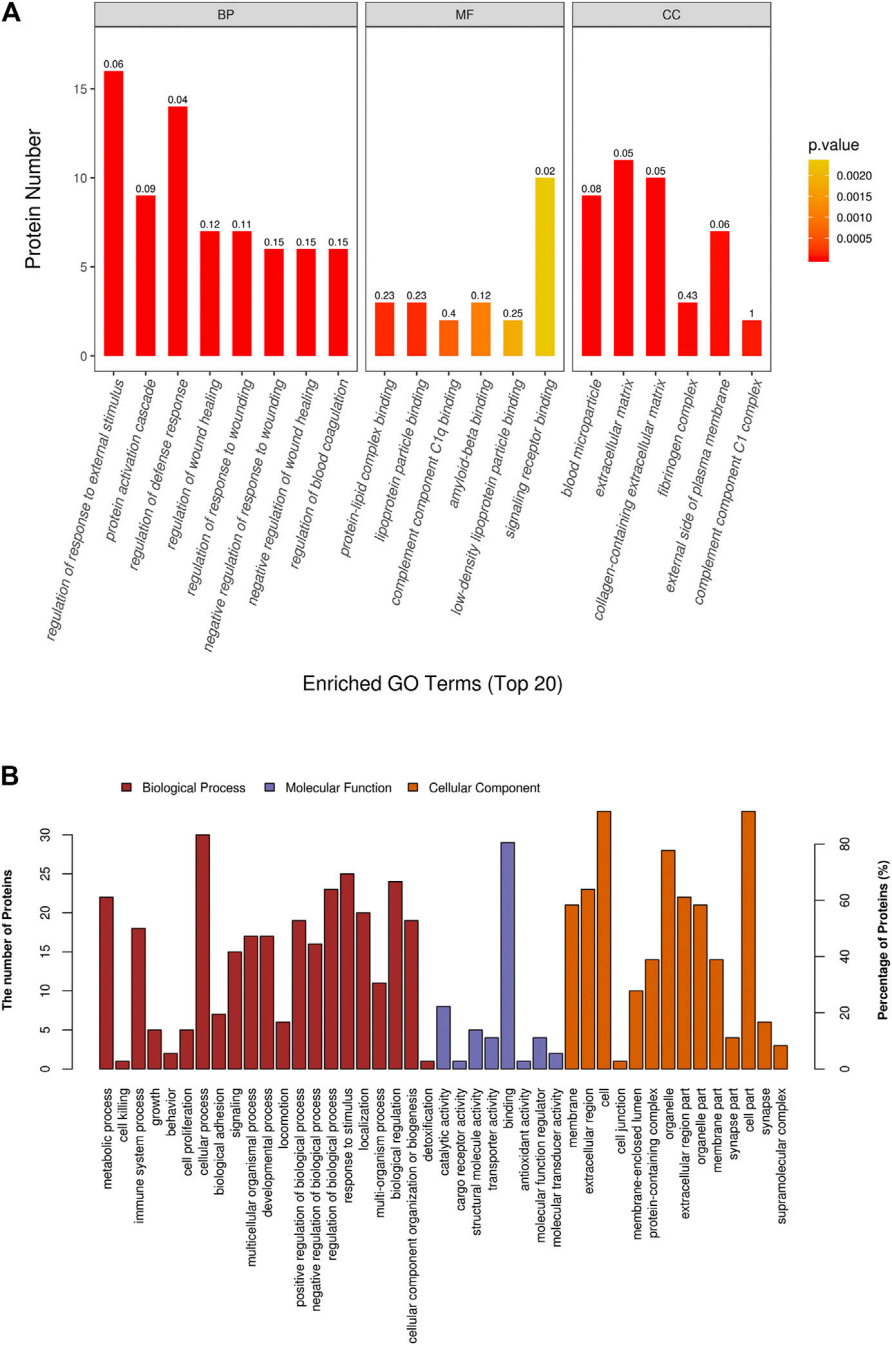
Gene ontology (GO) enrichment analysis of differential abundant proteins. **(A)** Top 20 enriched terms with smallest *p* values in GO enrichment analysis. **(B)** All level 2 GO terms involved in GO enrichment analysis.

GO functional annotation and enrichment analysis was performed for all DAPs, the results of which are shown in Supplementary Table S3. The GO terms were ordered according to the *p* value, and the top 20 enriched GO terms included eight terms in the biological pathway, six terms in the molecular function, and six terms in the cellular component. The biological pathway was mainly related to tissue refactoring and wound healing, including regulation of response to wound, regulation of wound healing, pathways that downregulate wound-related response and healing, response to external stimuli, and defense response. The molecular function was predominantly related to protein binding; protein–lipid complex binding and lipoprotein particle binding had the highest enrichment factor (0.23). For the cellular component, most DAPs were found in the extracellular matrix and some in organelles. Enrichment analysis results of the top 20 enriched GO terms with smallest *p* values are displayed in Figure 3A. GO terms at level 2 are on higher positions in the hierarchy of GO terms. Level 2 GO terms describe the properties of proteins more comprehensively, and all of the level 2 GO terms involved in GO enrichment are shown in Figure 3B. The metabolic process may occupy an important position in emphysematous phenotype–related biological pathways; besides, growth, cell proliferation, and developmental process were also involved. Binding was the most predominant molecular function of DAPs. In addition to the extracellular matrix, organelles may be the portion that DAPs were mainly distributed in.

**FIGURE 4:**
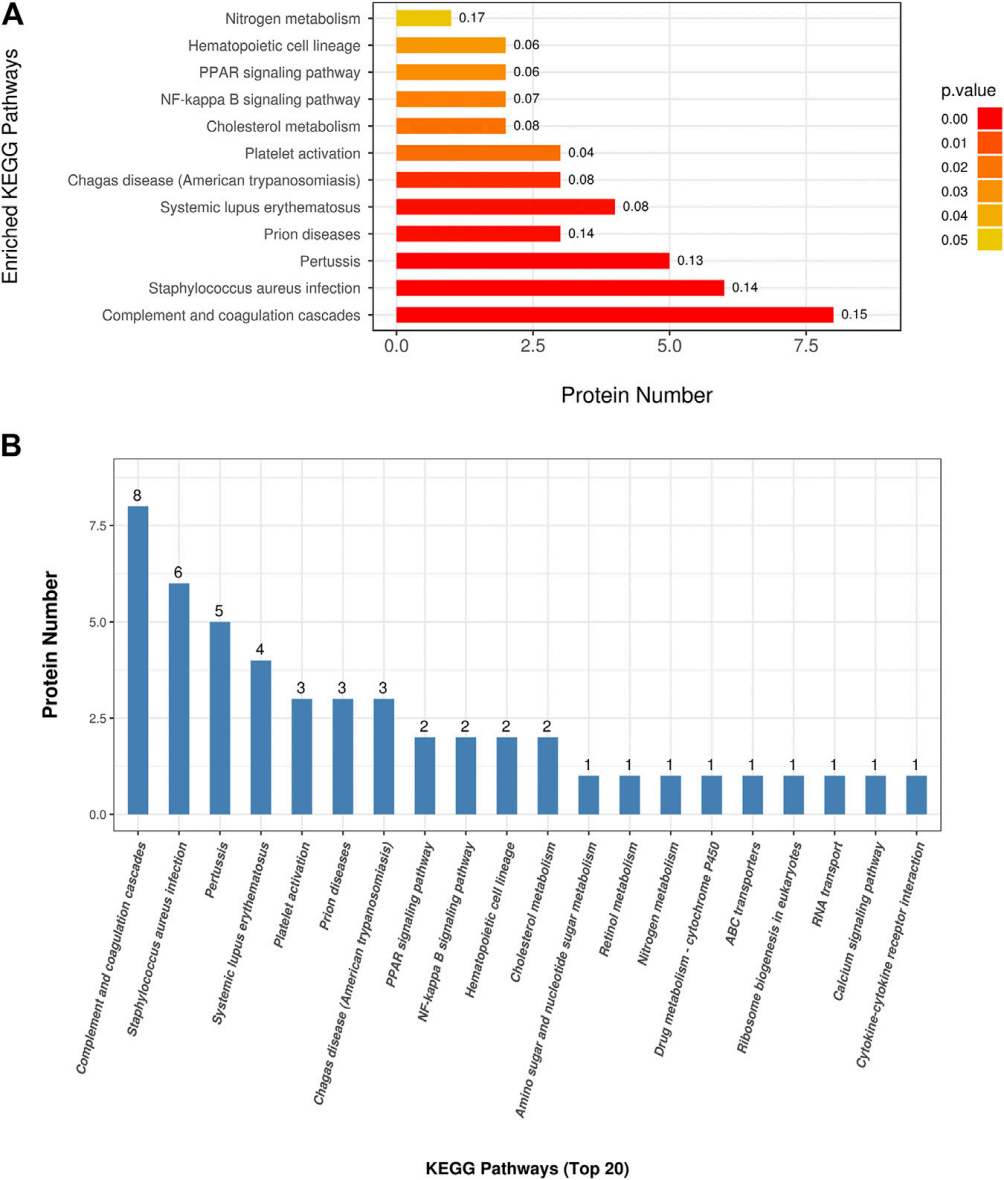
Kyoto Encyclopedia of Genes and Genomes (KEGG) enrichment analysis of differential abundant proteins. **(A)** Enriched terms in KEGG enrichment analysis with *p* values less than 0.05. **(B)** Top 20 KEGG terms with largest enriched protein numbers in KEGG enrichment results.

To understand the mechanisms and signaling pathways that DAPs may participate in the pathogenesis of COPD emphysematous phenotype, we performed KEGG pathway annotation and enrichment analysis of all DAPs; the specific results are shown inSupplementary Table S4. KEGG enrichment analysis showed that most DAPs were involved in metabolic and inflammatory pathways, including nitrogen metabolism, PPAR signaling, NF-κB signaling, and cholesterol metabolism. All KEGG terms with *p* values less than 0.05 are shown in Figure 4A and ordered according to the *p* value. The top 20 KEGG terms with largest enriched protein numbers are shown in Figure 4B.

**FIGURE 5:**
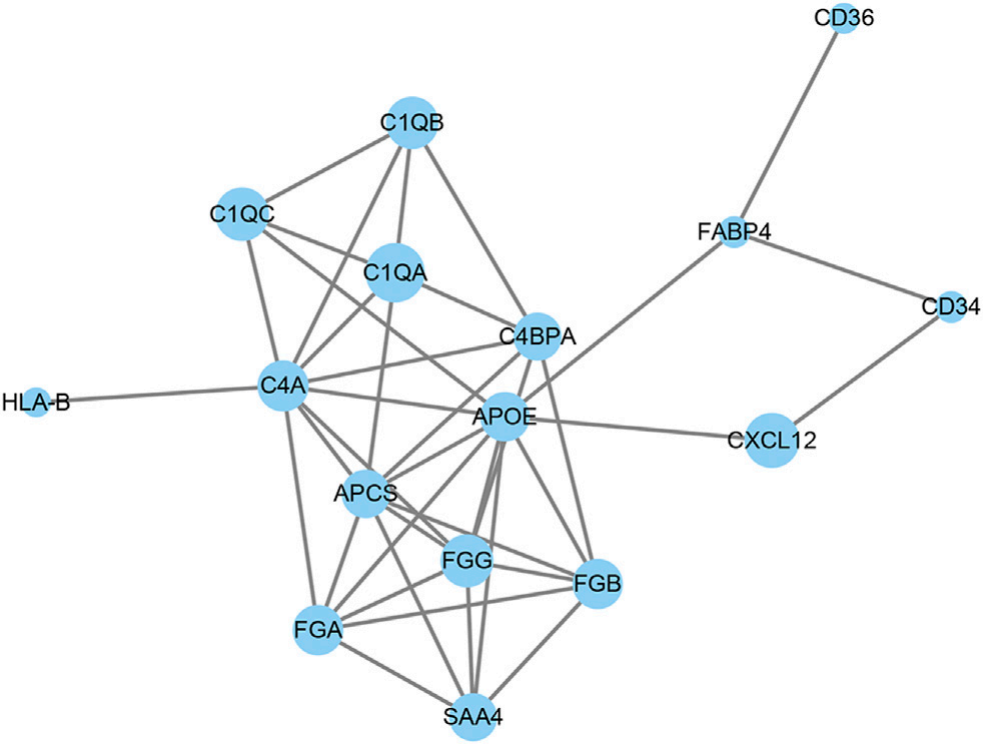
Protein–protein interaction (PPI) network of differential abundant proteins.

PPI network analysis was used to analyze the interaction between DAPs. As shown in Figure 5, 16 out of 36 DAPs had interaction nodes. Apolipoprotein E is the DAP that has most interactions with other DAPs, which indicates it may serve as a key molecule in the mechanism of emphysema. Besides, serum amyloid P-component, various chains of fibrinogen, and different subcomponent subunits of the complement interact with each other. These DAPs were related to the pathways in the results of GO and KEGG enrichment analyses. Therefore, these interactions may play roles in the pathogenesis of emphysematous phenotype, as well.

### Validation and Functional Annotation of DAPs

**FIGURE 6:**
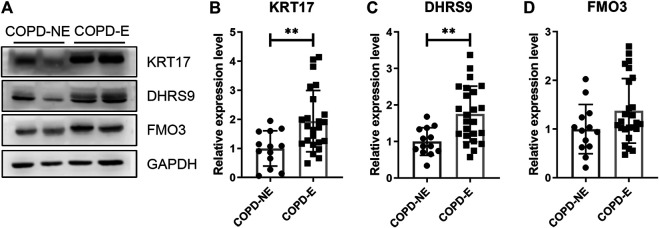
Type I cytoskeletal 17 (KRT17) and dehydrogenase/reductase SDR family member 9 (DHRS9) expressions are increased in the lung tissue samples of patients with chronic obstructive pulmonary disease emphysematous phenotype (COPD-E). **(A)** Western blotting results of KRT17, DHRS9, and FMO3 expressions in the lung tissue samples of COPD non-emphysematous phenotype (COPD-NE) and COPD-E groups. **(B-D)** Quantification of KRT17 (COPD-NE, *n* = 13; COPD-E, *n* = 24), DHRS9 (COPD-NE, *n* = 13; COPD-E, *n* = 24), and FMO3 (COPD-NE, *n* = 13; COPD-E, *n* = 25) protein expressions. **p < 0.01.

The expression of KRT17, dehydrogenase/reductase short-chain alcohol dehydrogenase/reductase (SDR) family member 9 (DHRS9), and dimethylaniline monooxygenase 3 (FMO3) was determined. Each lung tissue sample obtained from a subject was used as an independent sample in western blot. Western blotting results showing the expression of these proteins in the lung tissue samples of the validation cohort are shown in Figure 6. The protein expression of KRT17 (COPD-NE, *n* = 13, COPD-E, *n* = 24; *p* < 0.01) and DHRS9 (COPD-NE, *n* = 13, COPD-E, *n* = 24; *p* < 0.01) in the COPD-E group was higher than that in the COPD-NE group. However, the FMO3 expression was not significantly different between both groups. Western blotting results of all lung tissue samples are shown in Supplementary Figure S1.

The annotation results showed that KRT17 acts as a cytoskeletal protein and takes part in the process of cell growth and development, while DHRS9 participates in multiple metabolic pathways, such as retinol and steroid hormone biosynthesis (Table 5).

**TABLE 5:** Annotation of KRT17 and DHRS9.

Accession	Gene name	ID	Annotation term
Q04695	*KRT17*	GO:0070268	BP: cornification
		GO:0008544	BP: epidermis development
		GO:0031069	BP: hair follicle morphogenesis
		GO:0045109	BP: intermediate filament organization
		GO:0031424	BP: keratinization
		GO:0030307	BP: positive regulation of cell growth
		GO:0051798	BP: positive regulation of hair follicle development
		GO:0045727	BP: positive regulation of translation
		GO:0007165	BP: signal transduction
		GO:0071944	CC: cell periphery
		GO:0005829	CC: cytosol
		GO:0070062	CC: extracellular exosome
		GO:0005882	CC: intermediate filament
		GO:0045111	CC: intermediate filament cytoskeleton
		GO:0042289	MF: MHC class II protein binding
		GO:0032395	MF: MHC class II receptor activity
		GO:0005200	MF: structural constituent of cytoskeleton
		hsa05150	*Staphylococcus aureus* infection
		hsa04915	Estrogen signaling pathway
Q9BPW9	*DHRS9*	GO:0042904	BP: 9-cis-retinoic acid biosynthetic process
		GO:0008209	BP: androgen metabolic process
		GO:0030855	BP: epithelial cell differentiation
		GO:0042448	BP: progesterone metabolic process
		GO:0042572	BP: retinol metabolic process
		GO:0005789	CC: endoplasmic reticulum membrane
		GO:0030176	CC: integral component of endoplasmic reticulum membrane
		GO:0004022	MF: alcohol dehydrogenase (NAD) activity
		GO:0047044	MF: androstan-3-alpha,17-beta-diol dehydrogenase activity
		GO:0047023	MF: androsterone dehydrogenase activity
		GO:0016854	MF: racemase and epimerase activity
		GO:0004745	MF: retinol dehydrogenase activity
		GO:0047035	MF: testosterone dehydrogenase (NAD+) activity
		hsa00830	retinol metabolism

BP: biological pathway; CC: cellular component; MF: molecular function.

## Discussion

Patients with COPD emphysematous phenotype account for a considerable portion in clinics. Patients with this phenotype manifest progressive destruction of lung tissue and poor response to current pharmaceutical interventions of COPD (Janssen et al., 2019). With an aim of exploring the specific mechanism underlying the development of this phenotype, the proteome of lung tissues from patients with COPD emphysematous phenotype was studied using TMT quantitative proteomics. A total of 4,343 proteins were identified, out of which 36 were DAPs. Of these, 25 DAPs were upregulated and 11 DAPs were downregulated. This result was comparable with that of other proteomic analyses on lung tissue (Sun et al., 2019; Wu et al., 2020). Functional enrichment analysis of DAPs showed that tissue repair and cell proliferation and development were primarily related to the pathogenesis of emphysematous phenotype, in addition to inflammatory response, collagen disruption, and other well-recognized mechanisms in emphysema (Lan et al., 2019). Moreover, cholesterol-related, retinol-related, and other lipid–related metabolic pathways were enriched in KEGG pathway analysis. These results suggested that the impaired ability to repair injured tissue and lipid metabolism disorder might play an essential role in the development of prominent lung tissue destruction in COPD emphysematous phenotype. The differential expression of KRT17 and DHRS9 was validated in the lung tissue samples of patients with COPD emphysematous phenotype. Therefore, KRT17 and DHRS9 might affect the pathogenesis of COPD emphysematous phenotype by regulating tissue repair and lipid metabolism and act as phenotype-specific proteomic signatures.

Tissue repair is an essential process for all organisms to protect and maintain the normal structure and function. As structural destruction and collagen fibril disorder are prominently observed in patients with COPD emphysematous phenotype, the impaired ability to repair lung parenchymal and interstitial cells might play a significant role in its pathogenesis (Hu et al., 2020). The WNT/β-catenin pathway is a classical pathway involved in lung growth and development, and cell proliferation and differentiation (Königshoff and Eickelberg, 2010). Downregulation of this pathway will result in the retardation of tissue repair (Kneidinger et al., 2011; Uhl et al., 2015). Under normal conditions, WNT activates the JNK pathway by forming a complex with small G-proteins to promote cytoskeletal rearrangement during embryonic development (Wiggan and Hamel, 2002; Zhang et al., 2018). Moreover, the canonical WNT signaling pathway promotes the proliferation of type II alveolar epithelial cells (AECIIs) and *trans*-differentiation of AECIIs to type I alveolar epithelial cells (AECIs) that replenish injured ones in the damaged process of emphysema. In contrast, the non-canonical WNT signaling pathway inhibits proliferation and *trans*-differentiation processes (Skronska-Wasek et al., 2017). KRT17 is an important component of cytoskeleton. Functional annotation of DAPs showed that KRT17 participates in cell growth and development and cytoskeletal composition. Therefore, KRT17 has a potential to serve as an important target of the WNT/β-catenin signaling pathway. Both canonical and non-canonical WNT signaling pathways may rearrange or affect the abundance of cytoskeletal component—KRT17, resulting in the severe tissue destruction in patients with COPD emphysematous phenotype. Some studies have confirmed that the expression of cyclin D1, MMP7, c-Myc, and other vital targets of the WNT/β-catenin pathway is increased in KRT17-overexpressing cells (Wang et al., 2019). However, the mechanism through which canonical and non-canonical WNT signaling pathways interact with KRT17 warrants further studies, and the specific roles of KRT17 in the development of COPD and emphysema are still to be explored.

Retinoic acid is synthesized from retinol, and it regulates lung development and alveolar formation in the human body. Some studies have shown that retinoic acid can inhibit the activation of YAP signaling and epithelial–mesenchymal FGF signaling pathways, which promotes the proliferation and inhibits the differentiation of alveolar epithelial cells (Ng-Blichfeldt et al., 2018). In addition, retinoic acid can downregulate pro-apoptotic genes and inhibit the apoptosis of alveolar epithelial cells (Chatterjee and Chatterji, 2017). Therefore, the functional enrichment of DAPs in cholesterol and retinol metabolism in this study suggests aberrant proliferation and differentiation caused by impaired lipid metabolism. DHRS9 is a member of the SDR family. Crosstalk between DHRS9 and retinaldehyde dehydrogenase 1 (Raldh1) exerts important regulatory functions in the synthesis of retinoic acid. Unlike other members of the SDR family (e.g., Rdh10 and Rdh2), DHRS9 knockdown increased retinoic acid synthesis in RALDH1-overexpressing cells (Wang et al., 2011). Combined with the results of functional annotation and enrichment, DHRS9 can modulate the synthesis of retinoic acid, which in turn regulates the proliferation and differentiation of alveolar epithelial cells (Ng-Blichfeldt et al., 2018). Furthermore, clinical studies have confirmed that DHRS9 can regulate inflammation (Morichika et al., 2019) as well as the balance between MMP9 and TIMP1 (Mao et al., 2003) in cells. These results suggest that DHRS9 is predominantly involved in the pathogenic mechanism of emphysema in patients with COPD emphysematous phenotype, but specific molecular mechanisms are still unclear and remain to be explored.

Serum (Sridhar et al., 2019), expectorated sputum (Titz et al., 2015), and bronchoalveolar lavage fluid (Tu et al. 2014) samples have been used in previous proteomic studies to analyze the COPD-related proteome. However, the number of detected DAPs was relatively small and easily influenced by a systemic disease. Lung tissue is the direct affected site of COPD emphysematous phenotype. Therefore, lung tissue samples are the most suitable to identify key molecules involved in the mechanism underlying emphysema pathogenesis. To the best of our knowledge, this is the first proteomic analysis that focuses on emphysematous phenotype, to detect the specific DAPs in this phenotype.

There are some limitations to our study. Due to the fact that only few COPD patients were able to meet the inclusion criteria of our study, there are certain difficulties to collect lung tissue samples and the number of lung tissue samples in the validation cohort was relatively small. Moreover, there was an overlap between lung tissues from the discovery and validation cohorts. Furthermore, though wound repair and retinol metabolism may be the processes that KRT17 and DHRS9 participated in, they have not yet been demonstrated and explored in this study. In the future, more lung tissue samples from COPD patients should be involved and in-depth research studies should be conducted to explore the mechanism of COPD emphysematous phenotype development, including experiments *in vivo* and *in vitro*.

## Conclusion

A total of 36 DAPs were identified in lung tissue samples from patients in the COPD emphysematous phenotype group. Of these, 25 DAPs were upregulated and 11 DAPs were downregulated. KRT17 and DHRS9 were found to take part in the pathogenesis of emphysema. Moreover, wound repair and retinol metabolism may be the processes they participated. Therefore, KRT17 and DHRS9 can be used as phenotype-specific proteomic signatures in patients with COPD emphysematous phenotype.

## Data Availability

The original contributions presented in the study are publicly available. This data can be found here: ProteomeXchange Consortium (http://proteomecentral.proteomexchange.org), dataset identifier: PXD023900.
