# Supplementary material for: Identification of Proteomic Signatures in Chronic Obstructive Pulmonary Disease Emphysematous Phenotype
Source: Front Mol Biosci. 2021 Jul 1;8:650604. doi: 10.3389/fmolb.2021.650604 (PMC8280333; doi:10.3389/fmolb.2021.650604)
Supplement: Supplementary file 3 [file Table1.DOCX]

Supplementary Material

**Supplementary Table 1. CT scanning and reconstruction parameters of patients**

| ID | FOV  (mm) | Pixel size  (mm) | Reconstruction diameter (cm) | Number of slices | CT scanner |
| --- | --- | --- | --- | --- | --- |
| COPD-NE1 | 500.00 | 0.684 | 350 | 112 | Philips Brilliance iCT 256 |
| COPD-NE2 | 500.00 | 0.684 | 350 | 116 | Philips Brilliance iCT 256 |
| COPD-NE3 | 500.00 | 0.752 | 385 | 113 | Siemens SOMATOM Definition Flash |
| COPD-NE4 | 400.00 | 0.781 | 400 | 136 | TOSHIBA Aquilion ONE |
| COPD-NE5 | 500.00 | 0.782 | 400 | 133 | TOSHIBA Aquilion ONE |
| COPD-NE6 | 500.00 | 0.684 | 350 | 100 | Philips Brilliance iCT 256 |
| COPD-NE7 | 500.00 | 0.734 | 376 | 122 | Philips Brilliance iCT 256 |
| COPD-NE8 | 500.00 | 0.754 | 386 | 126 | Philips Brilliance iCT 256 |
| COPD-NE9 | 500.00 | 0.775 | 397 | 372 | Siemens SOMATOM Definition Flash |
| COPD-NE10 | 500.00 | 0.684 | 350 | 136 | Philips Brilliance iCT 256 |
| COPD-NE11 | 500.00 | 0.738 | 378 | 102 | Philips Brilliance iCT 256 |
| COPD-NE12 | 500.00 | 0.752 | 385 | 107 | Philips Brilliance iCT 256 |
| COPD-NE13 | 500.00 | 0.782 | 400 | 127 | Siemens SOMATOM Definition Flash |
| COPD-NE14 | 500.00 | 0.787 | 403 | 122 | Philips Brilliance iCT 256 |
| COPD-NE15 | 500.00 | 0.754 | 386 | 117 | Philips Brilliance iCT 256 |
| COPD-NE16 | 400.00 | 0.732 | 375 | 131 | TOSHIBA Aquilion ONE |
| COPD-E1 | 500.00 | 0.756 | 387 | 116 | Philips Brilliance iCT 256 |
| COPD-E2 | 500.00 | 0.770 | 394 | 112 | Philips Brilliance iCT 256 |
| COPD-E3 | 500.00 | 0.782 | 400 | 416 | TOSHIBA Aquilion ONE |
| COPD-E4 | 500.00 | 0.719 | 368 | 368 | Siemens SOMATOM Definition Flash |
| COPD-E5 | 500.00 | 0.740 | 379 | 382 | Philips Brilliance iCT 256 |
| COPD-E6 | 500.00 | 0.809 | 414 | 116 | Philips Brilliance iCT 256 |
| COPD-E7 | 500.00 | 0.703 | 360 | 153 | GE Optima CT660 |
| COPD-E8 | 500.00 | 0.719 | 368 | 100 | Philips Brilliance iCT 256 |
| COPD-E9 | 500.00 | 0.781 | 400 | 114 | Philips Brilliance iCT 256 |
| COPD-E10 | 500.00 | 0.787 | 403 | 121 | Siemens SOMATOM Definition Flash |
| COPD-E11 | 500.00 | 0.672 | 344 | 69 | Siemens SOMATOM Definition Flash |
| COPD-E12 | 400.00 | 0.781 | 400 | 125 | TOSHIBA Aquilion ONE |
| COPD-E13 | 500.00 | 0.754 | 386 | 124 | GE Optima CT660 |
| COPD-E14 | 500.00 | 0.760 | 389 | 134 | Philips Brilliance iCT 256 |
| COPD-E15 | 500.00 | 0.746 | 382 | 393 | Philips Brilliance iCT 256 |
| COPD-E16 | 500.00 | 0.723 | 370 | 115 | Siemens SOMATOM Definition Flash |
| COPD-E17 | 500.00 | 0.900 | 461 | 124 | Philips Brilliance iCT 256 |
| COPD-E18 | 500.00 | 0.684 | 350 | 128 | Philips Brilliance iCT 256 |
| COPD-E19 | 400.00 | 0.692 | 355 | 129 | TOSHIBA Aquilion ONE |
| COPD-E20 | 500.00 | 0.684 | 350 | 116 | Philips Brilliance iCT 256 |
| COPD-E21 | 500.00 | 0.782 | 400 | 131 | TOSHIBA Aquilion ONE |
| COPD-E22 | 500.00 | 0.782 | 400 | 128 | TOSHIBA Aquilion ONE |
| COPD-E23 | 500.00 | 0.782 | 400 | 129 | TOSHIBA Aquilion ONE |
| COPD-E24 | 500.00 | 0.748 | 383 | 117 | Philips Brilliance iCT 256 |
| COPD-E25 | 500.00 | 0.645 | 330 | 104 | Siemens SOMATOM Definition Flash |
| COPD-E26 | 500.00 | 0.747 | 383 | 118 | TOSHIBA Aquilion ONE |
| COPD-E27 | 500.00 | 0.773 | 396 | 152 | GE Optima CT660 |
| COPD-E28 | 500.00 | 0.893 | 457 | 151 | Philips Brilliance iCT 256 |
