# Supplementary material for: Identification of Proteomic Signatures in Chronic Obstructive Pulmonary Disease Emphysematous Phenotype
Source: Front Mol Biosci. 2021 Jul 1;8:650604. doi: 10.3389/fmolb.2021.650604 (PMC8280333; doi:10.3389/fmolb.2021.650604)
Supplement: Supplementary file 5 [file Image1.PDF]

Supplementary Material

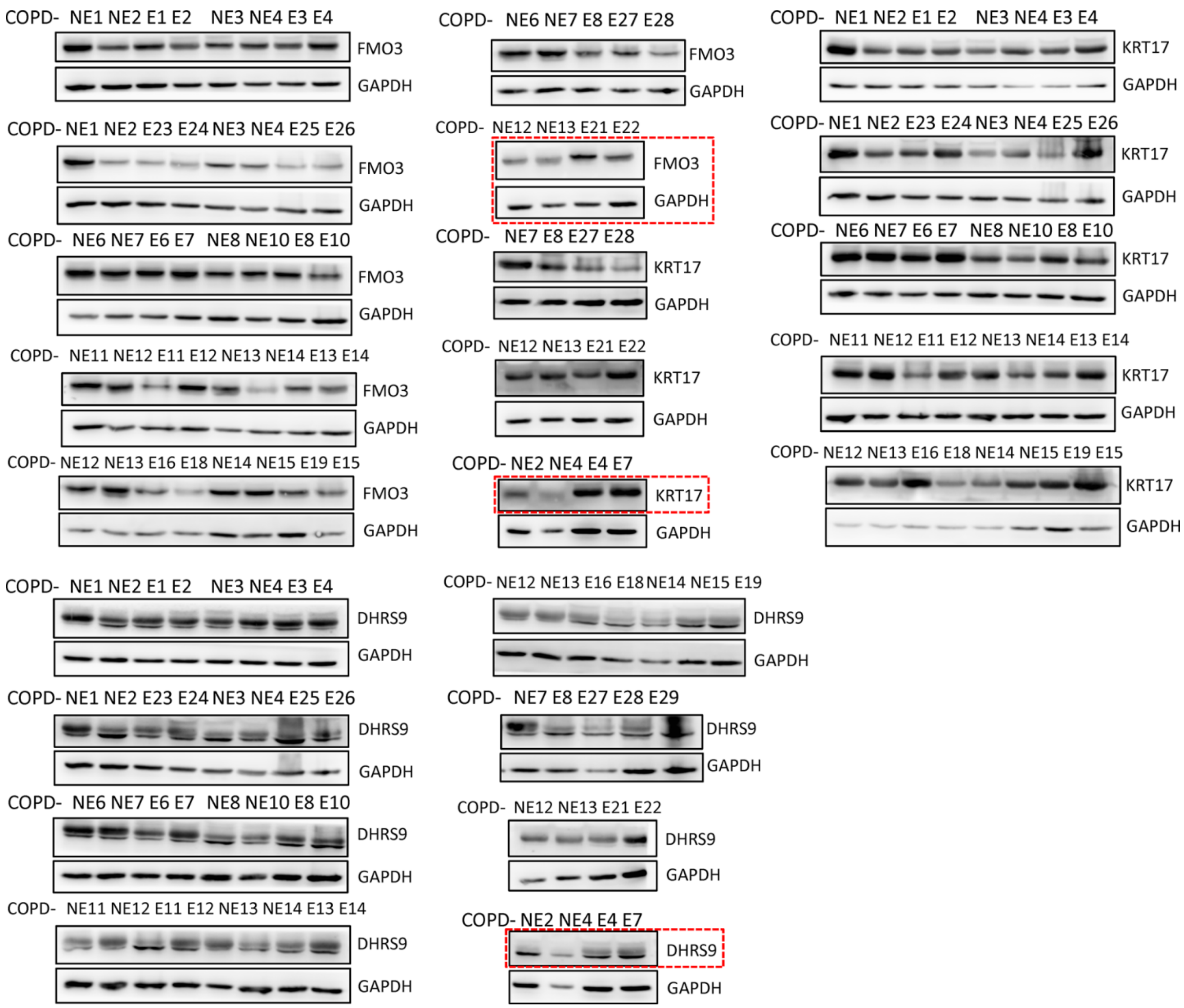

Supplementary Figure 1. Western blotting results of KRT17, DHRS9, and FMO3 expression in the all lung tissue samples of COPD non-emphysematous phenotype (COPD-NE) and COPD emphysematous phenotype (COPD-E) groups. The representative images used in figure 6A have been highlighted with red dotted lines.
